# Physical and mental illness comorbidity among individuals with frequent self-harm episodes: A mixed-methods study

**DOI:** 10.3389/fpsyt.2023.1121313

**Published:** 2023-03-09

**Authors:** Anvar Sadath, M. Isabela Troya, Sarah Nicholson, Grace Cully, Dorothy Leahy, Ana Paula Ramos Costa, Ruth Benson, Paul Corcoran, Eve Griffin, Eunice Phillip, Eugene Cassidy, Anne Jeffers, Frances Shiely, Íñigo Alberdi-Páramo, Katerina Kavalidou, Ella Arensman

**Affiliations:** ^1^School of Public Health, University College Cork, Cork, Ireland; ^2^National Suicide Research Foundation, University College Cork, Cork, Ireland; ^3^Kerry Primary Care Child, Adolescent and Family Psychology Service, Cork Kerry Community Healthcare, Health Service Executive, Kerry, Ireland; ^4^Liaison Psychiatry Services, Cork University Hospital, Cork, Ireland; ^5^Department of Psychiatry and Neurobehavioural Science, University College Cork, Cork, Ireland; ^6^Private Psychiatric Services, Dublin, Ireland; ^7^Health Research Board (HRB), Clinical Research Facility, University College Cork, Cork, Ireland; ^8^Hospital Clínico San Carlos, Madrid, Spain; ^9^National Clinical Programme for Self-Harm and Suicide-Related Ideation (NCPSHI), Health Service Executive, Dublin, Ireland; ^10^School of Applied Psychology, Australian Institute for Suicide Research and Prevention, Griffith University, Brisbane, QLD, Australia

**Keywords:** frequent self-harm, self-harm repetition, suicide intent, comorbidity, mental illness, physical illness, highly lethal self-harm

## Abstract

**Background:**

Research has indicated an increased risk of self-harm repetition and suicide among individuals with frequent self-harm episodes. Co-occurring physical and mental illness further increases the risk of self-harm and suicide. However, the association between this co-occurrence and frequent self-harm episodes is not well understood. The objectives of the study were (a) to examine the sociodemographic and clinical profile of individuals with frequent self-harm (regardless of suicidal intent) episodes and, (b) the association between physical and mental illness comorbidity, self-harm repetition, highly lethal self-harm methods, and suicide intent.

**Methods:**

The study included consecutive patients with five or more self-harm presentations to Emergency Departments across three general hospitals in the Republic of Ireland. The study included file reviews (*n* = 183) and semi-structured interviews (*n* = 36). Multivariate logistic regression models and independent samples *t*-tests were used to test the association between the sociodemographic and physical and mental disorders comorbidity on highly lethal self-harm methods and suicidal intent, respectively. Thematic analysis was applied to identify themes related to physical and mental illness comorbidity and frequent self-harm repetition.

**Findings:**

The majority of individuals with frequent self-harm episodes were female (59.6%), single (56.1%), and unemployed (57.4%). The predominant current self-harm method was drug overdose (60%). Almost 90% of the participants had history of a mental or behavioral disorder, and 56.8% had recent physical illness. The most common psychiatric diagnoses were alcohol use disorders (51.1%), borderline personality disorder (44.0%), and major depressive disorder (37.8%). Male gender (*OR* = 2.89) and alcohol abuse (*OR* = 2.64) predicted the risk of a highly lethal self-harm method. Suicide intent was significantly higher among those with a diagnosis of major depressive disorder (*t* = 2.43; *p* = 0.020). Major qualitative themes were (a) the functional meaning of self-harm (b) self-harm comorbidity (c) family psychiatric history and (d) contacts with mental health services. Participants described experiencing an uncontrollable self-harm urge, and self-harm was referred to as a way to get relief from emotional pain or self-punishment to cope with anger and stressors.

**Conclusion:**

Physical and mental illness comorbidity was high among individuals with frequent self-harm episodes. Male gender and alcohol abuse were associated with highly lethal self-harm methods. The mental and physical illness comorbidity of individuals with frequent self-harm episodes should be addressed *via* a biopsychosocial assessment and subsequent indicated treatment interventions.

## Background

Suicidal behavior is a leading cause of death and disability worldwide (1, 2). Although there are inconsistencies and varying definitions, suicidal behavior as a general term encompassing any suicidal thought or actions without taking additional steps to distinguish thoughts from plans, from non-fatal attempts, and from attempts that result in death (2). The term self-harm, used throughout this manuscript, refers to self-harm behavior regardless of suicidal intent. According to the Global Burden of Disease study 2019, self-harm was the main contributor to Years of Life Lost (YLL) from mental disorders in 31 European countries (1).

Self-harm is associated with multiple mental health conditions, including mood disorders (3–5), borderline personality disorder (BPD) (4, 6, 7), alcohol abuse, schizophrenia (5), adjustment disorder (3), eating disorders (7), and attention deficit hyper activity disorder (8). Emotional dysregulation, specifically a non-acceptance of emotional responses, lack of emotional awareness, difficulties in impulse control and engaging in goal-directed behavior, are all associated with self-harm (9). The act of self-harm has been described to be conducted with the intent to alleviate negative affect, and the negative affect and arousal are reduced by the performance of self-harm (10). Furthermore, studies that have examined self-harm severity/lethality in connection with mental disorders report that alcohol abuse (11, 12), BPD (13), and depression further increase the risk of more repeated highly lethal self-harm episodes (HLSMs) (14).

While the association between mental health conditions and self-harm is well established (15), the role of physical illness in self-harm or suicidal thoughts is less known. Previous research indicates that several chronic physical illnesses (5, 16, 17) including epilepsy, asthma, migraine, psoriasis, diabetes mellitus, eczema, and inflammatory polyarthritis are associated with an increased risk of self-harm (5). Suicide risk increases with the number of co-occurring physical illnesses (5). While a physical illness alone is not associated with self-harm or suicidal thoughts (15), the combination of physical and mental health conditions significantly increases the risk of both (15), with an elevated risk related to the onset of both physical and disorders occurring close in time to one another (18). Mental disorders, especially depression, often coexists among people with chronic physical illness (17, 19), and this is associated with heightened risk of self-harm. There is a wealth of studies in this area (20) albeit, specific to older people. The findings of a systematic review revealed that functional disability and numerous specific conditions (including malignant diseases, neurological disorders, pain, chronic obstructive pulmonary disease, liver disease, male genital disorders, and arthritis/arthrosis) were associated with self-harm among older persons (20).

Although mental and physical disorders are commonly reported among individuals who self-harm (5, 21), comorbidities specific to individuals with frequent self-harm episodes have rarely been studied. Yet, they are a high risk group due to the strong association between previous self-harm, future self-harm (22–26) and suicide (27, 28). The risk of self-harm repetition increases with each additional hospital self-harm presentation (23, 29), with the highest risk among those with a history of five or more presentations (30). Often referred to as major repeaters (30), these individuals represent a proportion of high suicide risk (31, 32). Individuals with frequent self-harm episodes comprise more than 10% of all self-harm presentations (29), frequently engage with health services (33) and have difficulties in controlling self-harm behavior, although the broader characteristics of this sub-group are under-researched (31, 32, 34). To our knowledge, only a few studies are available among this group (31, 35), and these studies included small samples of individuals with frequent self-harm episodes. These studies show that individuals with frequent self-harm episodes predominantly represent females and have a diagnosis of psychiatric illness (31, 35). Evidence is still lacking to conclusively provide a profile of these patients and associated comorbidities. Within this context, the main objectives of the present study were (a) to examine the sociodemographic and clinical profile of individuals with frequent self-harm episodes, (b) the association between physical and mental illness comorbidity and highly lethal self-harm methods (HLSMs), (c) the association between sociodemographic variables (age, gender, marital status, living and employment status) and HLSMs, (d) the association between suicide intent and psychiatric illnesses, and (e) to investigate the comorbidity factors contributing to self-harm repetition.

## Materials and methods

### Study design

**FIGURE 1:**
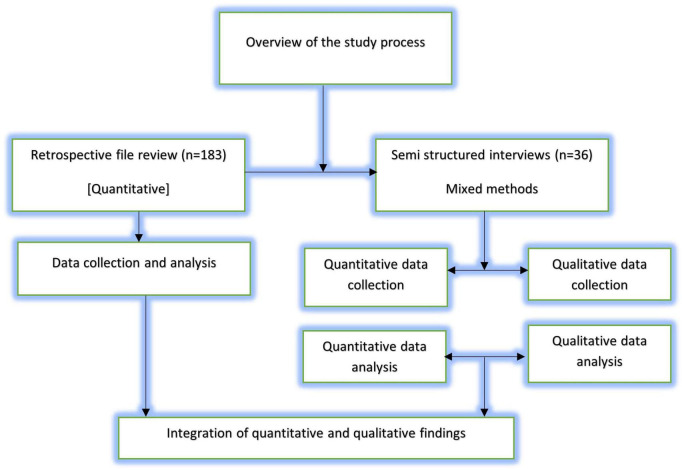
Overview of the study process.

This study uses a mixed-methods convergent parallel design in which quantitative and qualitative data were collected and analyzed simultaneously (36). The study included retrospective file review and semi structured interviews (Figure 1).

### Retrospective file review

All consecutive case files of individuals with frequent self-harm episodes from three general hospital Emergency Departments (ED) in the Republic of Ireland between March 2016 and July 2019 were reviewed.

Inclusion criteria: aged 18 and older, a history of five or more self-harm presentation to the ED, and alive on admission to the hospital following the self-harm episode. In each hospital, potential cases for review were first identified by hospital staff, i.e., Clinical Nurse Specialists or Crisis Nurses or Non-Consultant Hospital Doctors in the ED before the researchers were provided with access to the patients’ case files. The hospital staff were involved in screening self-harm cases according to the inclusion and exclusion criteria. The researchers conducted regular reviews of all self-harm cases to ensure that inclusion and exclusion criteria were being applied correctly and no cases were missed.

Data extraction was performed by the research team and included: medical history; psychiatric history; diagnosis; self-harm history; personal and family history; and sociodemographic information. Any relevant missing information in the files was coded accordingly by the research team.

Self-harm was defined as “an act with non-fatal outcome where an individual deliberately initiates a non-habitual behavior that without intervention from others will cause self-harm” (37). This included both acts with and without the intention to die. Frequent self-harm was defined as five or more previous self-harm presentations to hospital emergency departments, including the index presentation. Comorbidity was defined in the literature as “any distinct additional entity that has existed or may occur during the clinical course of a patient who has the index disease under study” (38). We operationally defined comorbidity as a co-occurring physical illness or mental disorder, or substance use disorder which existed with a self-harm episode among individuals with frequent self-harm episodes. We defined individuals with frequent self-harm episodes as those with a history of five or more self-harm presentations to the hospitals. We defined HLSMs in accordance with the criteria of Persett et al. (39), which includes hanging, strangulation, suffocation, cutting, submersion, firearm, jumping from a height or jumping in front of a moving object, fire and flames, and crashing of motor vehicles. Co-occurrence of any of these methods along with intentional drug overdose (IDO) was also recorded for this category. IDO in the absence of any other self-harm method was considered as non-HLSMs. Appendix 1 includes operationalization and dummy codes of the key independent (e.g., age, gender, marital status, BPD etc.) and dependent variable (HLSMs).

### Semi-structured interviews

From the consecutive individuals with frequent self-harm episodes cases identified through file reviews, a subgroup participated in semi structured interview study. The interviews and file reviews were conducted by trained members of the research team comprising of postdoctoral researchers, a PhD scholar, and a research officer, under the supervision of the principal investigator. Following interest from the patients, researchers subsequently provided a brief introduction of the research with an invitation letter and study information leaflet *via* post or in person whilst at the hospital premises. When patients agreed and consented to participation, the patient’s preferred time and venue were considered when scheduling the interview.

A semi-structured interview schedule, containing both closed and open-ended questions, was used for the interviews. The interview schedule covered standard sociodemographic data, medical and psychiatric history and psychosocial variables representing risk and protective factors for self-harm. The interview schedule used for this study was flexible, enabling the exploration of further details as required.

Suicide intent during the index self-harm presentation was assessed using the Beck Suicide Intent Scale (SIS) (40). The SIS is a fifteen-item interviewer-administered questionnaire, designed to assess the severity of suicidal intent associated with self-harm episodes. Each item scores 0–2, giving a total score range of 0–30, whereby a high score indicates high levels of suicide intent. The questionnaire is divided into two sections: the first eight items comprise the “circumstances” section, concerned with the objective circumstances of the self-harm act. The remaining seven items form the “self-report” section and are based on the patient’s own reconstruction of their feelings and thoughts at the time of the self-harm act. The SIS fifteen-item Cronbach’s alpha score in the current sample was 0.84.

### Ethical approval

The study complies with the Irish Data Protection Act of 1988 (41), the Irish Data Protection Amendment Act of 2003 (41) and General Data Protection Regulation (GDPR) 2018 (42). Ethical approval was obtained before the implementation of the GDPR. Following the implementation of GDPR, information leaflets and consent forms were updated to comply with GDPR and subsequently approved by the ethics committees. All participants included in the interview study provided written informed consent.

### Data analysis

#### Quantitative data analysis

The quantitative data collected through the file reviews and interviews were entered into Statistical Package for Social Sciences (SPSS) (version 26). Descriptive analyses were performed on data obtained from the file review and semi-structured interviews. Fisher’s exact test (FET) was used to compare the sociodemographic variables in both data sources. For all statistical tests, categorical variables were condensed into a binary response.

The association of sociodemographic variables, physical and mental disorders comorbidity and HLSMs was examined using FET and Odds Ratios with *p*-values and 95% confidence intervals provided. A multivariable logistic regression model was estimated introducing sociodemographic and physical and mental disorders comorbidity variables statistically significant in the FET. This variable selection procedure has the capability of retaining important confounding variables, resulting potentially in a richer model (43). The significant predictors from the first model (i.e., gender and alcohol use disorders) were further examined in an additional regression model for understanding the consistency of these predictors over two self-harm episodes. For the second model, “recent HLSMs” was treated as the dependent variable. All categorical variables were dummy coded (see Appendix 1). Further, we tested multicollinearity using linear regression and there was no evidence of this in the model (*variance inflation factor* ≤ 1.03).

An Independent *t*-test was used to test the association between physical and mental disorders comorbidity and suicide intent. The physical and mental disorders (depression, alcohol abuse, and chronic physical pain) were treated as grouping variables with two categories (yes/no) and SIS total score as a dependent variable. The SIS total score was normally distributed (*Shapiro–Wilk test* = *p* > 0.05).

#### Missing data handling

For the file review study, missing data occurred due to incomplete assessments. The missing data frequencies for different study variables ranged from *n* = 0–58. Missing data were not accounted for in the descriptive analysis. The percentages were calculated after exclusion of cases with missing values for the variable in question. Missing data were not replaced for the regression models, and the cases with missing data were excluded from the analysis on variable-by-variable basis.

#### Qualitative data analysis

Following the transcription of the interviews, thematic analysis was performed to identify themes related to physical and mental disorders comorbidity and self-harm. We followed the five key steps for conducting thematic analysis developed by Braun and Clarke (44).

The verbatim transcription was performed whereby the audiotaped interviews were transcribed into text. A list of codes was developed based on the reading of the transcripts, and these codes were allocated to the data, which helped to classify and stratify the data in a logical way, resulting in a proposed set of themes. The author (AS) performed the initial coding and analysis, and the themes were finalized in agreement with two other authors (IT and SN) who were involved in transcribing the interviews.

## Results

We identified 191 individuals with frequent self-harm episodes, and eight individuals were excluded due to incomplete data, resulting in 183 files being included in the file review study.

**FIGURE 2:**
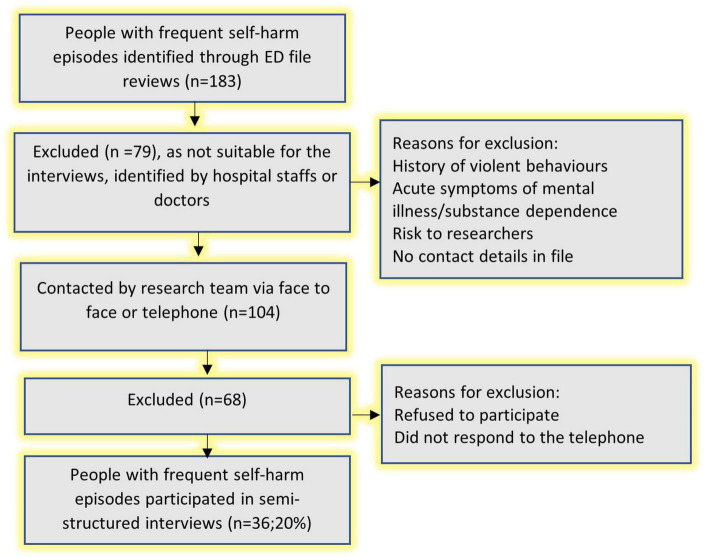
Recruitment of individuals with frequent self-harm episodes for semi-structured interviews.

From the consecutive cases identified through file reviews (*n* = 183), 79 (43%) were not approached for the semi-structured interviews. They were either deemed not suitable for interviews by hospital staff or consultant doctors due to acute symptoms (e.g., severe psychotic or mood symptoms), or were unconscious or in induced coma because of the severity of the self-harm act, history of violence and aggressive behavior, risk to researchers or their contact details were missing. The remaining patients (*n* = 104) were contacted by the research team *via* telephone or *via* face-to-face contact during their hospital visits to invite them to participate in the semi-structured interview study. However, 68 patients did not participate as they either refused (*n* = 22) or were never answered our phone or phone disconnected (*n* = 46). A total of 36 respondents participated in the semi-structured interview study, representing 20% of the file review sample (Figure 2).

The interviews ranged from 1 h and 10 min to 4 h and 18 min. Of the 36 participants interviewed, 31 agreed to (audio) record the interviews. For the other participants, we took notes on their responses. The data collection was completed between March 2016 and September 2019.

Table 1 shows the comparison of study participants in file review and interviews, and there was no significant difference between the two groups regarding sociodemographic characteristics.

**TABLE 1:** Sociodemographic and self-harm characteristics of the study participants.

		File review (*n* = 183) frequency (%)	In-depth interviews (*n* = 36) frequency (%)	FET *P*-value
**Gender**				
	Male	74 (40.4)	11 (30.6)	0.295 (NS)
	Female	109 (59.6)	25 (69.4)	
**Age**				
	Below 35	91 (49.7)	13 (36.1)	0.260 (NS)
	35 or above	92 (50.3)	23 (63.8)	
**Marital status**				
	Single	101 (56.1)	24 (66.7)	0.560 (NS)
	Married/Co-habiting/Long-term relationship	56 (31.1)	12 (33.3)	
	Widowed/Divorced/Separated	23 (12.7)		
	*NR/Unknown	3		
**Living arrangements**				
	Alone	43 (24.3)	7 (19.4)	0.370 (NS)
	With family of origin/Spouse/Children	104 (59.7)	21 (58.3)	
	Others (e.g. friends)	27 (15.5)	7 (19.4)	
	NR/Unknown	9		
**Employment status**				
	Employed	23 (13.7)	7 (19.4)	0.317 (NS)
	Unemployed	96 (57.4)	6 (16.7)	
	Long term disability	25 (14.9)	19 (52.7)	
	Fulltime student	16 (9.5)	3 (8.3)	
	Other (e.g., house wife)	7 (4.1)	1 (2.8)	
	NR/Unknown	16		

Categories condensed for the test (see Appendix 1); FET, fisher’s exact test; NR, not recorded in the file; NS, not significant.

As shown in Table 2, the majority of participants had engaged in IDO in their current (61.3%) and recent (67.3%) self-harm episodes.

**TABLE 2:** Self-harm characteristics of the participants in file review study.

		*n* (%)
**Mode of presentation**		
	Brought in by ambulance	100 (61.7)
	Self-presented	33 (20.3)
	Brought in by family/Partner/Others	21 (12.9)
	General Practitioner (GP) referral	9 (5.5)
	Not recorded (NR)/Unknown	20
**Method of self-harm (index)**		
	Drug overdose	111 (61.3)
	Hanging/Strangulation/Suffocation	12 (6.6)
	Cutting	49 (27.0)
	Submersion (drowning)	4 (2.2)
	Other (fire and flames/Crashing motor vehicle)	5 (2.7)
	NR/Unknown	2
*Method of self-harm (recent)		
	Drug overdose	101 (67.3)
	Hanging/Strangulation/Suffocation	10 (6.7)
	Cutting	32 (21.3)
	Submersion (drowning)	3 (2.0)
	Other (firearm, jumping from high place/Jumping in front of a moving object)	4 (2.6)
	NR/Unknown	33
**Multiple methods of self-harm**		
	Yes	15 (8.3)
	No	166 (92.7)
	NR/Unknown	3
**Alcohol consumed at the time of self-harm**		
	Yes	87 (55.0)
	No	71 (44.9)
	NR/Unknown	25
**Number of previous self-harm episode**		
	Five or more	29 (15.8)
	10 or more	153 (83.6)

*Recent self-harm refers the most recent self-harm episode documented in the file prior to the index episode.

Table 3 displays physical and mental disorders comorbidity and treatment details of the study participants. As the table indicates, around 90% of the respondents were diagnosed with any mental or behavioral disorders and 56.8% had a physical illness in the recent past. Among the participants with a physical illness, almost all (96.7%) had a diagnosis of a mental/behavioral disorder.

**TABLE 3:** Mental disorder, physical illness, and treatment details of the participants in file review study.

Diagnosis	Yes (%)	No (%)	Within any physical illness	
			**Yes (%)**	**No (%)**
Any mental disorders	164 (89.6)	12 (6.6)	118 (96.7)	4 (3.3)
*Any recent physical illness	71 (56.8)	54 (43.2)		
**Diagnosis**				
BPD/EUPD	66 (44.0)	84 (66.0)	53 (46.5)	61 (53.5)
Major depressive disorders	56 (37.8)	92 (62.1)	39 (35.8)	70 (64.2)
BPAD	20 (13.6)	126 (86)	10 (8.8)	104 (91.2)
Psychosis/Schizophrenia	10 (6.7)	138 (93.2)	7 (6.0)	110 (94.0)
**Anxiety disorders	37 (25.6)	107 (74.3)	22 (19.5)	91 (80.5)
Eating disorder	6 (4.2)	135 (95.7)	5 (4.5)	105 (95.5)
Alcohol use disorders	86 (51.1)	82 (48.8)	52 (44.8)	64 (55.2)
Drug use disorders	65 (34.8)	102 (61.1)	40 (33.9)	78 (66.1)
**Treatment**				
Previous mental health treatment (inpatient)	98 (72.0)	38 (28.0)		
Previous mental health treatment (outpatient)	135 (92.4)	11 (7.5)		
Previous treatment with addiction services	48 (38.0)	78 (61.9)		
Current psychiatric medications	139 (83.7)	27 (16.2)		

Not recorded/missing values were ranged from 15 to 58 across the categories. *Includes epilepsy, appendix, asthma, stroke, COPD, gallstone, diabetics, encephalitis, Parkinsonism, kidney infection, optic neuritis, and malignant syndrome. BPD/EUPD, borderline/emotionally unstable personality disorder; BPAD, bipolar affective disorder. **Anxiety disorders included phobic anxiety disorders (F40) and other anxiety disorders (F41), obsessive compulsive disorder (F42) and reaction to severe stress, and adjustment disorders (F43) categories.

### Physical illness, traumatic life events, and family history of mental disorders among individuals with frequent self-harm episodes in interview study

Among the participants (total *n* = 36), the majority had chronic physical pain in the past year (72.2%), one-fourth of the participants had asthma (25%), more than one-fifth had orthopedic (19.4%), or metabolic problems (22.2%). More than half of the participants had a recent reduction in physical capabilities (55.6%) and 63.9% were on medication for a physical illness. The participants had history of various traumatic life events including, violent sexual assault (77.8%), directly witnessing a sudden death (22.2%), sudden death of a loved one (63.9%) severe bullying or torture (55.6%), and humiliation or loss of face (19.4%). Many participants had family history of self-harm/suicide (47.2%), mental illness (63.9%), substance abuse (69.4%), and violent behavior (55.6%).

### Association of sociodemographic, physical, and mental disorders comorbidity with the use of lethal self-harm methods in the index self-harm act and in the most recent self-harm act

Gender, employment status and alcohol use disorders were significantly associated with index HLSMs in FET, and these variables were subsequently entered into multivariate binary logistic regression model (see Table 4). The Omnibus tests of model coefficients was significant (χ^2^ = 17.63; *df* = 3; *p* = 0.001) while the Hosmer and Lemeshow test was not significant (*p* = 0.726), indicating that the model fitted the data well. Overall, the model explained 14.6% (Nagelkerke R^2^) of variance on the index HLSMs. Male gender (*X*^2^ = 8.45; *P* = 0.004; *OR* = 2.89) and alcohol use disorders (*X*^2^ = 7.32; *P* = 0.007; *OR* = 2.64) increased the likelihood of a HLSMs. Employment status was not significant (*P* > 0.05) when adjusted for confounders.

**TABLE 4:** Association of sociodemographic and comorbidity variables with the use of highly lethal self-harm methods in the index self-harm act and in the most recent self-harm act.

	HLSMs in index self-harm act					HLSMs in most recent self-harm act
	**Yes (*n* = 80)**	**No (*n* = 101)**	**FET *p*-value**	**Unadjusted OR (CI)**	**Adjusted OR (CI)**	**Adjusted OR (CI)**
**Sociodemographic**						
Age (35 years or above)	46.3% (37)	52.5 % (53)	0.455	ns		
Gender (male)	51.2% (41)	30.7% (31)	0.001	2.97 (1.69–4.36)	2.89 (1.41–5.91)	2.32 (1.08–4.95)
Marital status (single)	56.4% (44)	56% (56)	1.00	ns		
Living arrangements (alone)	28.6% (26)	21.1 (20)	0.287	ns		
Employment status (not working)	81.7% (58)	65.3% (62)	0.023	2.97 (1.83–4.95)	ns	
**Comorbidities**						
Depressive disorder	41.7% (25)	34.4% (31)	0.391	ns		
Psychosis/Schizophrenia	9.7% (06)	4.6% (04)	0.320	ns		
Anxiety disorders	31.1% (19)	20.7% (17)	0.176	ns		
Alcohol use disorders	63.8% (44)	42.3 (41)	0.005	2.40 (1.27–4.53)	2.64 (1.30–5.34)	3.65 (1.69–7.87)
BPD/EUPD	50.8% (32)	39.1% (34)	0.183	ns		
Physical illness (Recently)	60.8% (31)	54.8% (40)	0.581	ns		

Multivariable model for index act: Omnibus tests of model coefficients = χ^2^ = 17.63; df = 3; *p* = 0.001; Hosmer and Lemeshow test- *p* = 0.726; Cox and Snell *R*^2^ = 0.108; Nagelkerke *R*^2^ = 0.146. Dependent variable has 2 missing values. Multivariable model for most recent act: Omnibus tests of model coefficients = χ^2^ = 16.74; df = 2; *p* = 0.000; Hosmer and Lemeshow test *p* = 0.897; Cox and Snell *R*^2^ = 0.114; Nagelkerke *R*^2^ = 0.158. Dependent variable has 33 missing values; FET, fisher’s exact test; CI, confidence interval.

We further conducted a multivariate binary logistic regression model to validate the previously identified predictors (i.e., gender and alcohol use disorders) by treating the recent HLSMs as a dependent variable (see Table 4). The Omnibus tests of model coefficients was significant (χ^2^ = 16.74; *df* = 2; *p* = 0.000) while the Hosmer and Lemeshow test was not significant (*p* = 0.899), indicating that the model fit that data well. Overall, the model explained 15.8% (Nagelkerke R^2^) of variance on a recent HLSMs. Male gender (*X*^2^ = 4.73; *P* = 0.030; *OR* = 2.32) and alcohol use disorders (*X*^2^ = 10.89; *P* = 0.001; *OR* = 3.65) increased the likelihood of a recent HLSMs (see Table 4).

### Suicide intent

The mean score of the SIS was 17 (*SD* = 6.48), indicating mild to moderate suicide intent during the index self-harm. For the subdomains, “circumstances around the self-harm act,” the eight-item mean score was 6.74 (*SD* = 2.86), which indicated mild suicide intent while “thoughts and feelings at the time of self-harm act,” the seven-item mean score was 9.96 (*SD* = 4.41), indicating moderate suicide intent.

An independent sample *t*-test was conducted to compare suicide intent of those with a diagnosis of depressive disorder, personality disorder, alcohol use disorders, and chronic physical pain. There was a significant difference in suicide intent score between those with a diagnosis of depressive disorder (*M* = 19.15; SD = 4.08) and those without this diagnosis (*M* = 14.58; *SD* = 6.95); (*t* = 2.43; *p* = 0.020) (Table 5).

**TABLE 5:** Association of physical and mental illness comorbidity and suicide intent among individuals with frequent self-harm episodes participated in the interview study (*n* = 36).

Diagnosis	Frequency	Mean (SD)	*t*	Sig
**Depressive disorder**				
Yes	19	19.15 (4.08)	2.43	0.020
No	17	14.58 (6.95)		
**Personality disorder**				
Yes	21	16.95 (6.65)	0.055	0.956
No	15	17.06 (5.17)		
**Alcohol use disorders**				
Yes	17	18.41 (6.63)	1.350	0.186
No	19	15.73 (5.23)		
**Chronic physical pain**				
Yes	26	17.50 (6.15)	0.801	0.428
No	10	15.70 (5.69)		

Dependent variable = suicide intent scale total.

### Qualitative findings

Thematic analysis resulted in four themes associated with self-harm and physical and mental disorders.

#### The functional meaning of self-harm

Self-harm was described as an uncontrollable urge especially those with a diagnosis of BPD. The self-harm act was used to get relief from emotional pain. And while intention to die was low among individuals with frequent self-harm episodes with BPD, self-harm acts often resulted in hospitalization and moderately lethal injuries.

*Actually, it’s a big word to say, “I want to die.” With me, it’s not really that I want to die. I just want problems and pain to stop*…… *It’s like, you know, trying to tell myself not to do it, knowing a part of me wanted to do it. And then just at that moment was like an explosion for me after everything that happened with my friends, and then my mom’s birthday and calling into work, and then my boyfriend left me*… *(Female, BPD)*.

Many others described self-harm as a means of self-punishment, in which anger toward another person was oriented to themselves as form of self-punishment.

*I didn’t want to die, I felt I just needed to punish myself for what the council had done to me, I had no other way out, I couldn’t harm anyone else so I had to harm myself*…. *I brought satisfaction on myself for somebody else for what they had done (Female, BPD).*

Self-harm urges often were precipitated by multiple psychosocial stressors. There was a sudden outbreak of multiple stressful life events prior to the self-harm act. As these demands were perceived excessive, participants felt helpless, and the self-harm act occurred as a way of coping.

In contrast, self-harm occurred in some cases, without report of any acute stressors.

*I used to cut and burn just here and there every couple of months. I used to just get this urge and used to just kind of a comfort and I don’t even know why I used to do it. One day I was just sitting at home, having a great time with everyone and I just went into the bathroom and I was calm and relaxed, and I cut just for the sake of doing it like and cover them up and went out and sat down 5 min later got up and went out and did the same thing and just came out laughing a joker like nothing happened*…… *(Female, BPD)*.

Medication often helped to improve mood and reduce self-harm urge. However, when the self-harm urge became stronger, some patients stopped taking their medication and engaged in self-harm.

*In March I stopped the medication because I knew that I didn’t want to have control anymore, I wanted to be destructive to myself, I wanted to harm myself, I wanted to do it, I felt I felt I wanted to (Female, BPD).*

#### Comorbidities in self-harm

Many participants with BPD had a diagnosis of depressive episode or a recurrent depressive disorder. And also, they had a chronic physical health condition or physical pain. In addition, multiple substance misuse was also very common, and most participants had alcohol consumption prior to their self-harm episode.

*I am an alcoholic, I use cannabis, cocaine, and prescribed medication as well. I started the drug use from the age of 15*…. *I was under the influence of both alcohol and cannabis at the time of self-harm*…. *I don’t remember how much I had (referring quantity of alcohol) (female, BPD and MDD).*

*I had been off cannabis for most of last year. When I was in Canada, and I kind of wanted to keep things chiller in Christmas and family time, so I started taking Cannabis again, in December. I did drink and smoke cannabis on the day (referring the day of the index self-harm) (Female, BPD).*

Participants described the impact that alcohol use and misuse had on their self-harming behavior. In a few cases, participants used alcohol to get rid of traumatic flashbacks and to maintain sleep. Many described an increased likelihood of self-harming and more severe self-harm acts following alcohol use.

*I lost my nephew, who hang himself on a tree. All images and memories of this like. I get nightmares and I couldn’t sleep. I’m just constantly waking up see my nephew hanging on a tree. I started go on drinking (alcohol) to get rid of this, which escalated the problem even more like. I felt so down myself, I didn’t know what to do*…*I just gave up my life basically. I did the same (hanging), but the rope snapped (Male, MDD).*

Physical illness, especially chronic physical pain and a reduction in physical capabilities were described as issues impacting participants prior to the self-harm episodes. For some, the pain and reduction in physical capabilities were associated with a depressive episode. Some participants described unexplained somatic symptoms and these symptoms often coexisted with a diagnosis of MDD.

*I feel like every day there’s something (pain), you know, whether it’s my stomach or my head. I have low energy and kind of just random pains*…. *If I took painkillers I just had that bit of energy, and that bit of energy to get stuff done, get up, and get into library, study, and be able to go to the gym things like that (Female, MDD).*

For many participants, reduced physical capabilities and physical pain were described in relation to multiple physical health conditions.

*My thyroid had completely given up*… *I was just getting more ill and had no energy, my body was just shutting down (Female, BPD).*

*I had a prolapsed disc which was operated on*… *I have back pain, it’s a kind of chronic condition so that’s going on more than 12 months*… *I go through phases when it gets really bad, I go on painkillers (Male, BPAD).*

While experience of physical illness or physical pain was not described as having a direct link to self-harm episodes, these conditions often co-existed with a psychiatric illness such as depressive disorder or bipolar affective disorder or borderline personality disorder, and these together heightened the risk of self-harm. For instance, experience of unbearable physical pain and a pessimistic view that there is no solution to this problem (due to depression) often triggered to self-harm episodes.

#### Family psychiatric history

In many cases, participants described the existence of multiple mental illnesses or behavioral disorders in their families. Specifically, a family history of substance use disorders, depression, or self-harm/suicide were most described.

*My mom, dad, and my other brother are alcoholic. My mom had depression, my dad had depression and anger management issues. My other brother and my sister had attention deficit hyperactivity disorder (Female, MDD, and BPD).*

*Every one of my family see psychiatrists. They all take antidepressants or some type of sleeping tablet or something to keep them going (Female, BPD).*

*My uncle hang himself suffered mental illness, and my mom’s mother, she killed herself. My dad is an alcoholic (Female, BPD).*

Family psychiatric history contributed to self-harm mainly in two ways. First, individuals with frequent self-harm episodes were vulnerable to self-harm due to the significant family history. Second, prescribed drugs were available in the home for the treatment, which increased the risk for intentional overdoses.

#### Contacts with mental health services

Since most individuals with frequent self-harm episodes had been suffering with significant mental health difficulties, they were required to continue treatment with healthcare services. For mental health issues, participants preferred contacting the specialized mental health services rather than their General Practitioner (GP). However, most participants reported substantial difficulties in accessing of help when they were in need. Most participants contacted mental health services when they experienced low mood or self-harm urges. When they experience delays in accessing support, it has led to worsening of mood symptoms and or increasing self-harm urges, which subsequently resulted in self-harm episodes.

*Appointment is in 3 months. If you need an appointment sooner, it’s very hard. But it’s hard enough to get an appointment (Female, MDD).*

*I am waiting for an appointment with a psychologist for a long period*… *I am still waiting. They have told me this is (waiting period) is usual because only one psychologist is available (Male, BPD).*

Nevertheless, some participants were able to get help from a private therapist or private psychiatrist.

*There is such a long waiting list (hospitals), I’m happy that I found someone (psychotherapist) but if there is someone not so confident to look for help themselves, then they might not even be here anymore at this stage (Female, BPD).*

## Discussion

**FIGURE 3:**
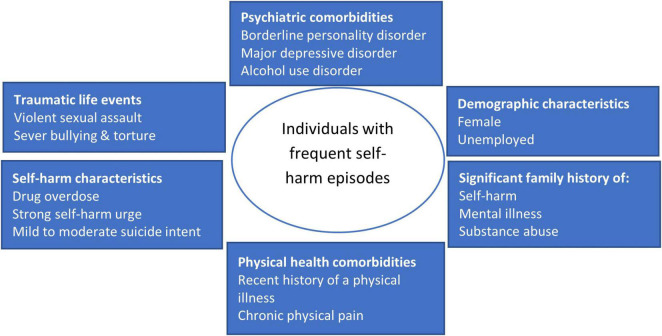
Key profile of individuals with frequent self-harm episodes.

The study’s findings are in line with the emerging academic and clinical discourse that individuals with frequent self-harm episodes are a self-harm subgroup with a distinct sociodemographic and clinical profile. The findings are consistent with two other studies in this area, indicating that individuals with frequent self-harm episodes are predominantly female, have a psychiatric diagnosis, (31, 35) are unemployed (31), have a diagnosis of BPD and/or substance abuse, and have a high personal and familial risk history (31, 35). We provide further information on this subgroup: the predominant self-harm method was IDO, they reported a history of recent physical illness and physical pain, a history of many traumatic life events including violent sexual assault, severe bullying, and torture, with an extensive family history of mental illness, self-harm and substance abuse, and they expressed mild to moderate suicide intent. Physical (5) and psychiatric co-existing disorders are known to be associated with self-harm (5, 23). However, the proportion of individuals with frequent self-harm episodes suffering from BPD, MDD, substance abuse, physical illness, physical pain, sexual assault was substantially high in our study. For instance, we have found high prevalence of chronic pain (72.2%) and/or major depressive disorders (37.8%) among individuals with frequent self-harm episodes as compared to the prevalence of 35.5% (chronic pain) (45) or depressive disorders (22.8%) (46) reported in primary care population (45) or in general community population (46). Moreover, many of these conditions coexisted each other and constituted to an extensive comorbidity profile (Figure 3).

The IDO as a predominant self-harm method among individuals with frequent self-harm episodes could be explained in the context of psychiatric illness and treatment. For instance, most of the individuals with frequent self-harm episodes or their family members were on treatment for a psychiatric illness, or physical pain, thus increasing the risk by stock piling prescribed medication. It is likely that accessibility and availability of the drugs, combined with strong self-harm urges increase the risk of IDOs. This is in line with previous research indicating that the prescription of psychotropic drugs is associated with the use of these drugs in IDOs, particularly minor tranquillizers (47).

Although most individuals with frequent self-harm episodes had engaged in IDO, we examined the characteristics of the subgroup representing those with HLSMs. Male gender and alcohol use disorders consistently predicted HLSMs, while other sociodemographic or physical and mental disorders variables were not significant. The association between gender and self-harm methods has been documented previously, with male gender increasing the risk of having a violent (39, 48, 49) or severe self-harm episodes (50, 51). Violent methods were further associated with risk of suicide (52). Studies also reported an association between substance abuse and violent (53) or lethal self-harm (54). Inconsistent with other studies, (55, 56), a coexisting psychosis was not associated with HLSMs in the current study. However, notably, the prevalence of psychosis was relatively lower in the study samples. A diagnosis of MDD was not associated with HLSMs, while this was associated with suicide intent. The association between MDD and suicide intent is well established (57–59). Although a higher proportion of individuals with frequent self-harm episodes had physical illness, this was not associated with HLSMs. The association between a physical illness and HLSMs is under researched. Nevertheless, some available research has demonstrated the association of physical illness with self-harm repetition (5), although this was also inconsistent (60). Physical pain was reported by most of the study participants, but this was not associated with suicide intent. A few other studies had reported the association between pain intensity and suicide ideation (61) and pain-related condition and suicide (62). However, the association between physical illness or physical pain and self-harm is under-researched (23), and requires more research to provide reliable conclusions.

The qualitative findings reveal critical themes including functional meaning of self-harm, self-harm comorbidity, family psychiatric history, and contacts with mental health services. Most of the participants who engaged in repeated self-harm had low intention to die and the self-harm often resulted from an uncontrollable self-harm urge, as reported in previous research (63). Previous researchers have even developed instruments to measure the cognitive and emotional aspects of this craving (64) and repeated self-harm behaviors have been conceptualized as an addiction (32, 34). Only one study examined self-harm craving among individuals with frequent self-harm episodes, and reported characteristics similar to addictive behaviors including tolerance, loss of control, and continuation of self-harm behaviors despite significant negative consequences (34). The findings of the current study support the notion that individuals with frequent self-harm episodes engage in addictive behaviors relating to self-harm, mostly coexisting with BPD. An uncontrollable self-harm urge or some addictive behaviors are mostly suited to explain the repeated self-harm behavior in BPD but is insufficient to explain the repeated episodes of other individuals with frequent self-harm episodes who presented without this diagnosis. It is unlikely that a single conceptualization like self-harm urges, or addiction would be adequate to explain these complex behaviors. Thus, it is postulated that this subgroup has an extensive profile of self-harm comorbidity evidenced by the qualitative and quantitative findings. Among individuals with frequent self-harm episodes with a physical illness, almost all had a diagnosis of a mental/behavioral disorder, a finding supported by previous research demonstrating that a number of chronic diseases are associated with suicidal thoughts and suicide attempts (15, 65). Congruent to the current findings, high personal and family risk history including sexual abuse was also reported among individuals with frequent self-harm episodes (35, 66). We also identified mental health system related barriers for accessing help, which indeed has potential impact on self-harm and wellbeing. Many system related issues including lack of mental health professionals and delays in availing of appointments with public mental health services had been identified previously, with a recommendation for effective system planning (67).

This study has many strengths, including the focus on individuals with frequent self-harm episodes who have the highest risk of prospective repeated self-harm. Hence, examining their unique profile and associated comorbidities is important for effective assessment and management of self-harm. Among this subgroup, this is the first mixed-method study involving a large sample size. Participants in the semi-structured interview group can be considered a strong representation of individuals with frequent self-harm episodes while there were no significant differences between the two groups on key sociodemographic characteristics. The file review and interview data complemented each other to address the study objectives. For example, physical pain, suicide intent, traumatic life events and family history of mental disorders were not documented well in the file review, while the semi structured interview data had provided more comprehensive information. Similarly, the file review data addressed the potential predictors of HLSMs, while the qualitative data reflected participants’ own experiences of having physical and mental health comorbidities related to self-harm. A further strength of this study is that the potential predictors identified were consistent over multiple self-harm episodes within one individual. Therefore, these variables may be considered to be more strongly associated with HLSMs. Highly lethal methods of a recent self-harm episode are known to be related to a high risk of subsequent suicide (68), which underlines the significance of these findings for suicide prevention.

Nevertheless, the study findings must be interpreted with the following considerations. First, we concurrently used file review data and semi-structured interview data to examine the study objectives. Although this approach has potential to complement each other, the file review data is retrospective in nature, hence, the limitations applicable to retrospective findings are applicable to the current findings. Second, many variables in the file review had unrecorded values, which were not included for the analysis, while this would have impacted on our prevalence estimates. This under recording points out the need for improving biopsychosocial assessment and documentation for this self-harm subgroup. Third, the diagnosis of a physical or mental disorder was based on the casefiles of the patients, made by a registered physician or psychiatrist. While the diagnosis was relied on the individual clinician’s judgment, we did not use any standard instruments to verify the diagnosis with the subgroup participated in the interview study. Fourth, certain groups of patients could not be considered for the study (e.g., patients with acute psychotic or mood symptoms or those who were unconscious or in induced coma because of the severity of the self-harm act). While their condition implied inability to take part in the interview study and or provide informed consent, valuable lived experience was missed from this group. Fifth, the operationalization of highly lethal or violent self-harm methods varied, and this could include (39) or exclude (69) self-cutting. We considered self-cutting as HLSM as there are large scale longitudinal studies which demonstrate that self-cutting increases the risk of suicide, compared to those with self-poisoning (26, 70). Although a HLSM can increase the risk for suicide, the method alone can’t be completely attributed to suicide or even lethality or severity. A serious suicide attempt is a combination of medical lethality, potential lethality of the method used, and severity of the objective circumstances of the suicide intent (71).

## Recommendations

Biopsychosocial assessment should be carried out to all individuals with frequent self-harm episodes as a part of routine clinical services in EDs. Due to incompleteness of biopsychosocial assessments in 30% of participants, it is recommended to improve training and supervision to ensure completeness of core elements of the biopsychosocial assessment, which is line with recommendations of the National Clinical Programme for Self-harm and Suicide related Ideation in Ireland (72), Royal College of Psychiatrists (73) and NICE guideline (74). Screening of possible comorbidities should be introduced on the clinical practices, specifically, screening for substance use disorder, borderline personality disorder and major depressive disorders. Physical illnesses especially chronic physical pain also should be assessed and managed. While the association between physical and mental health comorbidities and self-harm repetition is evident, future studies should examine the specific association between these comorbidities and completed suicide.

In addition, future studies should also identify the effective components of psychotherapeutic interventions (e.g., biopsychosocial risk assessment/safety planning/cognitive behavior therapy) for individuals with frequent self-harm episodes and or training and capacity building programmes for key healthcare professionals for addressing the increased self-harm and suicide risk among this self-harm subgroup.

## Conclusion

This is one of few studies examining mental and physical health comorbidities among a high-risk self-harm group. The findings demonstrate that almost all individuals with frequent self-harm episodes had a diagnosis of mental disorders, and two thirds had a diagnosis of physical pain, indicating a high level of physical and mental disorders comorbidity. This study underlines the need to consider individuals with frequent self-harm episodes as a self-harm subgroup with a unique clinical and comorbidity profile, with the requirement for these patients to receive a biopsychosocial assessment after each episode of self-harm.

## Data availability statement

The raw data supporting the conclusions of this article will be made available by the authors, without undue reservation.

## Ethics statement

The studies involving human participants were reviewed and approved by the Clinical Research Ethics Committee of the Cork University Teaching Hospitals [reference number EMC 4(2) 12/04/16] and the HSE Mid-Western Regional Hospital Research Ethics Committee (reference number REC 018/6). The patients/participants provided their written informed consent to participate in this study.

## Author contributions

AS, MT, SN, GC, DL, AR, PC, and EA: conceptualization. AS, MT, SN, GC, DL, AR, EA, and ÍA-P: methodology. AS, MT, SN, GC, DL, AR, and EA: investigation. AS and EA: writing—original draft. MT, SN, GC, DL, AR, RB, PC, EG, EP, EC, AJ, FS, ÍA-P, KK, and EA: writing—review and editing. EA: funding acquisition and supervision. EA, EC, and AJ: resources. All authors contributed to the article and approved the submitted version.

## Appendix

**APPENDIX 1:** Operationalization and dummy codes of the study variables.

Variables	Description and transformation of the variables
Age	Age collected as continuous variable. Categorized to binary response (Dummy codes: Below 35 years = 1; 35 years or above = 0).
Gender	Operationalized as either male or female (Dummy codes: male = 1; female = 0).
Marital status	We condensed the subcategories and divided the patients either being single or in relationships. The first category included only those who were single. The second category included those who were married, cohabiting, or in short-term/long-term relationships, widowed, divorced, or separated (Dummy codes: single = 1; in relationship = 0).
Living arrangements	We condensed the subcategories and divided the patients as either being living alone or living with others. Living with others included living with spouse, family of origin, children or with friends (Dummy codes: living alone = 1; living with others = 0)
Employment Status	We condensed the subcategories and divided the patients as either currently working, or not working. The first category included those who were engaged in any amount of paid or unpaid employment, student, housewife; and the later included those who were unemployed or on long term disability (Dummy Codes: currently not working = 1; currently working = 0).
Highly lethal self-harm Methods (HLSMs)	We categorize the self-harm method as HLSMs or non-HLSMs. The first category included hanging, strangulation, suffocation, cutting, submersion, firearm, jumping from high place or jumping in front of a moving objects, fire and flames and crashing motor vehicles. Coexistence of these methods along with drug overdose (i.e., multiple method) was also coded for this category. The second category included only drug overdose (Dummy codes: Yes, HLSMs = 1; No = 0).
Depressive disorders	This section included all of the major depressive disorders - depressive episode, recurrent depressive disorder, depressive episode/disorder with mild, moderate, severe intensity, with or without psychotic symptoms (Dummy codes: Yes, diagnosed a depressive disorder = 1; No = 0).
Psychosis/Schizophrenia	A diagnosis of any psychosis spectrum disorders were included in this category (Dummy codes: Yes, diagnosed with a psychosis spectrum disorder = 1; No = 0).
Anxiety disorders	A diagnosis of any anxiety spectrum disorders were included in this category (Dummy codes: Yes, diagnosed with a anxiety spectrum disorder = 1; No = 0).
Alcohol use disorder	Any alcohol use disorder with mild, moderate or severe intensity were included in this category (Dummy codes: Yes, diagnosed an alcohol use disorder = 1; No = 0).
Borderline personality disorder (BPD)/Emotionally unstable personality disorder (EUPD)	A diagnosis of BPD/EUPD were included in this category (Dummy codes: Yes, diagnosed with a BPD/EUPD = 1; No = 0).
Physical illness	A recent diagnosis (past 1 year period) of any physical illnesses were included in this category (Dummy codes: Yes, diagnosed to have physical illness = 1; No = 0).
